# Suppressive effects of induced pluripotent stem cell-conditioned medium on *in vitro* hypertrophic scarring fibroblast activation

**DOI:** 10.3892/mmr.2014.3115

**Published:** 2014-12-18

**Authors:** YE REN, CHEN-LIANG DENG, WEI-DONG WAN, JIANG-HONG ZHENG, GUANG-YU MAO, SONG-LIN YANG

**Affiliations:** Department of Plastic Surgery, Shanghai Jiaotong University Affiliated Sixth People’s Hospital, Shanghai 200233, P.R. China

**Keywords:** hypertrophic scarring, induced pluripotent stem cell, conditioned medium, fibroblast activation

## Abstract

Hypertrophic scarring (HS) is a type of fibrosis that occurs in the skin, and is characterized by fibroblast activation and excessive collagen production. However, at present, therapeutic strategies for this condition are ineffective. Previous studies have identified that the mutual regulation of chronic inflammation, mechanical force and fibroblast activation leads to the formation of HS. Induced pluripotent stem cells (iPSCs) are novel bioengineered embryonic-like stem cells, initially created from mouse adult fibroblasts. The current study demonstrated that iPSC-conditioned medium (iPSC-CM) may significantly suppress hypertrophic scar fibroblast activation. It was observed that in the presence of iPSC-CM, the level of collagen I was markedly reduced and α-smooth muscle actin, a marker for myofibroblasts (activated fibroblasts that mediate mechanical force-induced HS formation), exhibited a significantly lower level of expression in human dermal fibroblasts (HDFs) activated with transforming growth factor-β1. Additionally, iPSC-CM attenuated the local inflammatory cell response by blocking the adhesion of human acute monocytic leukemia cell monocytes and fibroblasts *in vitro*. In addition, the contractile ability of HDFs may be reduced by iPSC-CM. These observations suggest that iPSC-CM may protect against processes leading to hypertrophic scarring by attenuating fibroblast activation, blocking inflammatory cell recruitment and adhesion and reducing the contractile ability of fibroblasts.

## Introduction

There are multiple conditions in which damage leads to fibroblast activation and excessive collagen production, which can result in fibrosis of various tissues (1). For example, fibroproliferative disorders occurring following dermal trauma may lead to hypertrophic scarring (HS). HS results from abnormal and excessive deposition of extracellular matrix (ECM) during skin wound healing, particularly collagen I and III, in different proportions depending upon the type of tissue wounded and the age of the individual (2). Collagen metabolism is crucial to scar formation and determines its properties (3). In addition, HS is characterized by fibrosis and inflammation, which is associated with several inflammatory cytokines and growth factors affecting fibroblast activity, including transforming growth factor β-1 (TGF-β1); fibroblast growth factor; platelet-derived growth factor; macrophage-derived growth factor; interleukin-1 and tumor necrosis factor-α (4). By modulating levels of these proteins, HS formation may be attenuated or prevented. However, there are currently no effective therapeutic methods for HS that prevent fibroblast activation.

Induced pluripotent stem cells (iPSCs) are novel bioengineered embryonic-like stem cells (5) that were initially created from mouse adult fibroblasts with four factors (Oct3/4;Sox2; Klf4; and *c*-Myc) by optimizing retroviral transduction (5). A previous study demonstrated that with either iPSC transplantation or iPSC-conditioned medium (iPSC-CM) injection, interstitial and vascular fibrosis may be significantly inhibited (6). Additionally, iPSCs have previously been suggested to be effective for the treatment of myocardial (6,7), pulmonary (8) and renal (9) fibrosis.

These observations support the hypothesis that iPSCs may suppress HS fibrosis by inhibiting fibroblast activation. Although iPSCs have the ability to differentiate into cell types of the three germ layers, it is difficult to manage the direction of this differentiation. iPSCs cannot be maintained in an undifferentiated state by a simple alteration in culture medium, and previous studies have demonstrated that they may develop into tumors, lose their self-renewal capacity or lose the potential to differentiate into the cell type required for therapeutic transplantation *in vivo* (8,10). One study observed that the therapeutic effects of iPSC-CM are similar to iPSCs in lung injury, and act via a similar signaling pathway (11). Therefore, the current study aimed to determine whether iPSC-CM is able to inhibit fibroblast activation, by examining fibroblast-associated properties, including activation, contraction and adhesion to human acute monocytic leukemia (THP-1) cells in cultured human skin fibroblasts.

## Materials and methods

### Cell culture and conditioned medium

iPSCs were generated from embryonic fibroblasts of C57/B6 mice and were provided as a gift by Dr. Kazutoshi Takahashi (Institute for Frontier Medical Sciences, Kyoto University, Kyoto, Japan). The iPSCs were reprogrammed by the transduction of retroviral vectors encoding four transcription factors, Oct-4, Sox2, *c*-Myc and Klf4, and cultured in iPSC medium to maintain an undifferentiated state, as previously described (12). Human dermal fibroblasts (HDFs) were isolated from normal human foreskin. All primary human fibroblasts were obtained from each sample prior to tissue fixation in 10% formalin (Nanchang Yulu Co., Jianxi, China) for routine histological examination. The tissue sections were cut into 1–3-mm cubes and incubated with 200 U/ml type I collagenase (Worthington Biochemical Corporation, Lakewood, NJ, USA) for 4 h at 37°C. The fibroblast cell cultures were maintained in Dulbecco’s modified Eagle’s medium (DMEM; 11965-092) supplemented with 10% fetal bovine serum (FBS), 2 mM glutamine, 100 U/ml penicillin and 100 mg/ml streptomycin (all from Gibco Life Technologies, Grand Island, NY, USA). THP-1 cells (American Type Culture Collection, Manassas, VA, USA) were maintained in RPMI 1640 medium (Gibco Life Technologies) supplemented with 10% FBS, 100 U/ml penicillin, 100 mg/ml streptomycin and 0.5 mM/l β-mercaptoethanol (Gibco Life Technologies). All cell lines were incubated at 37°C in a humidified incubator with 5% CO_2_ and cells from passages 6–8 were used. Conditioned medium from iPSCs (2×10^5^ cells/cm^2^) was diluted to 50, 30 and 0% by HDF-conditioned medium (HDFs-CM).

A total of 15 foreskin samples were collected from the Shanghai Jiaotong University Affiliated Sixth People’s Hospital (Shanghai, China) following approval by the ethics committee for human studies. The patients provided informed consent, and none had a systemic disease or had been previously treated for scars.

### Total protein synthesis assay

Following treatment with 0, 30, 50 or 100% iPSC-CM for 24 h, 4×10^5^ cells HDFs were harvested. The total protein was determined by the microplate bicinchoninic acid method using the BCA Protein Assay kit (Pierce Biotechnology, Inc., Rockford, IL, USA) in accordance with the manufacturer’s instructions.

### Cell adhesion assay

Cell adhesion assays were perfomed as described previously (13). HDFs were seeded at a density of 3×10^5^ cells/well into 24-well plates until confluence was reached, then were incubated with DMEM supplemented with 10% FBS, 2 mM glutamine, 100 U/ml penicillin and 100 mg/ml streptomycin. THP-1 cells were maintained in RPMI 1640 medium, supplemented with 10% FBS, 100 U/ml penicillin, 100 mg/ml streptomycin and 0.5 mM/L β-mercaptoethanol for 24 h and then were labeled fluorescently using 2.5 mM Cellstain-calcein-AM-solution (Dojindo Molecular Technologies, Inc., Kumamoto, Japan). THP-1 and HDF cells were co-cultured with the THP-1 suspension at a concentration of 5×10^5^ cells/ml and 0, 30, 50 or 100% iPSC-CM was added into each well for 3 h. The plates were centrifuged at 134 × g for 3 min and subsequently incubated at 37°C for 60 min. The medium and the nonadherent THP-1 cells were removed and each well was washed with phosphate-buffered saline three times. Adherent THP-1 cells were then microscopically quantified at a magnification of ×100 in four random visual fields for each well, and were subsequently imaged using an Axiovert 200 inverted fluorescence microscope (Zeiss, Oberkochen, Germany).

### Three dimensional (3D) collagen gel contraction assay

HDFs were seeded into 32-mm bacteriological plates (density, 6×10^4^ cells/ml; 2 ml/dish) in DMEM supplemented with 10% FBS, 100 U/ml penicillin and 100 mg/ml streptomycin, sodium ascorbate (50 mg/ml; Gibco Life Technologies) and 0.3 mg/ml acid-extracted collagen I from newborn calf skin (IBFB Pharma GmbH, Leipzig, Germany), as previously described (14), with 0, 30, 50 or 100% iPSC-CM. Furthermore, the contraction efficiency of the iPSC-CM was compared between quiescent and activated HDFs treated with TGF-β1 (Sigma-Aldrich, St. Louis, MO, USA). The cells were cultured at 37°C for 60 min to allow collagen polymerization to occur. The gels were then released from the plates by tilting them slightly. Gradual gel contraction was assessed by measuring the gel area at four time points, including 6, 12, 18 and 24 h. The data are presented as the mean ± standard error of three independent experiments, each conducted in triplicate.

### RNA isolation and reverse transcription-quantitative polymerase chain reaction (RT-qPCR)

Total RNA was isolated from cultured HDFs using TRIzol reagent (Invitrogen Life Technologies), and the integrity of the RNA was determined by 2% UltraPure agarose gel (Gibco Life Technologies) electrophoresis (15). For RT-qPCR, 2 μg total RNA was reverse transcribed at 37°C for 1 h in a 25-μl reaction medium containing 250 mM Tris-hydrochloric acid (HCl), 375 mM potassium chloride (KCl), 15 mM magnesium chloride (MgCl_2_), 50 mM dithiothreitol, 10 mM deoxynucleotide triphosphates (dNTPs), 0.5 μg oligo (dT) 20 primer, 100 U reverse transcriptase (M-MLV) and 25 U ribonuclease inhibitor (all from Takara Bio, Inc., Otsu, Japan) and were subjected to PCR amplification with the primers described in Table I. Glyceraldehyde 3-phosphate dehydrogenase (GAPDH) was amplified as the internal control. The RT products (0.5-l.0 μg) were amplified with 1 U Taq DNA polymerase (Takara Bio, Inc.) and 1 mM of each primer in a 50 μl reaction mix containing 50 mM KCl, 10 mM Tris-HCl, 1.5 mM MgCl_2_ and 0.02 mM each of four dNTPs as follows: Initial denaturation for 3 min at 94°C, 30 cycles of amplification, 1 min of denaturation at 94°C, annealing temperatures of 57°C and 53°C for the collagen Iα1 and GAPDH primers, respectively, 1 min of extension at 72°C and a final 5-min elongation period at 72°C. Parallel PCR assays without reverse transcriptase were performed for each sample to confirm that the PCR products resulted from cDNA rather than from genomic DNA. The PCR products (10 μl) were analyzed by 2% agarose gel electrophoresis (15). The relative abundance of mRNA was calculated by densitometric analysis using Digital Science 1D Image Analysis software, version 3.0 (Kodak, Rochester, NY, USA).

### Western blotting

Cells were lysed with radio-immunoprecipitation assay lysis buffer (Beyotime Institute of Biotechnology, Jiangsu, China) supplemented with 1 mM phenylmethylsulfonyl fluoride (Adamas-Beta, Ltd., Shanghai, China). The cell lysates were subject to western blot analysis, which was conducted as described in previous studies (15). The primary antibodies used were as follows: Polyclonal rabbit anti-human α-SMA IgG (1:500; ab15263; Abcam, Cambridge, UK) and monoclonal mouse anti-human collagen I IgG1 (1:200; sc-59772; Santa Cruz Biotechnology, Inc., Dallas, TX, USA).

### Statistical analysis

Statistical analysis was performed using SPSS, version 13.0 (SPSS, Inc., Chicago, IL, USA) and a paired samples t-test was used to identify any differences between the groups. P<0.05 was considered to indicate a statistically significant difference.

## Results

### Cytotoxicity of iPSC-CM

**Figure 1:**
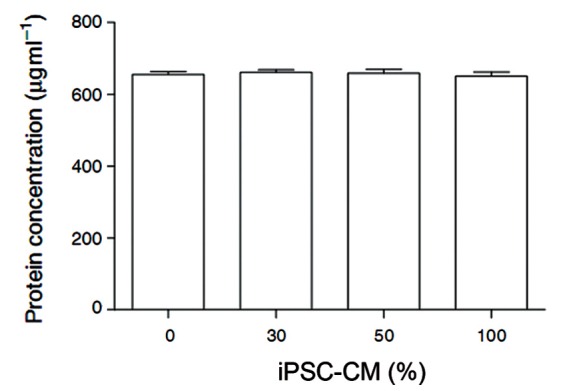
Total protein level of HDFs following treatment with 0, 30, 50 and 100% iPSC-CM for 3 days, n=3. All values are presented as the mean ± standard error. HDFs, human dermal fibroblasts; iPSC-CM, induced pluripotent stem cells-conditioned medium.

To evaluate the cytotoxicity of iPSC-CM on fibroblasts, the total protein products following treatment of HDFs with 0, 30, 50 or 100% iPSC-CM for 24 h were measured. No significant differences were observed in the total protein among the four groups, which suggests that total fibroblast activity is not markedly affected by iPSC-CM (Fig. 1).

### Suppressive effect of fibroblast activation by iPSC-CM

HS is formed by the abnormal accumulation of ECM. This predominantly consists of collagen, particularly type I collagen, which is produced by fibroblasts and is vital to HS formation. The proliferative stage of HS is marked by proliferation and activation of fibroblasts, thus, the level of type I collagen may be a marker of fibroblast activation. Myofibroblasts (activated fibroblasts) are identified by α-SMA expression and stress fiber formation (16), and their recruitment, retention and differentiation are commonly triggered by local stimuli of the microenvironment. Those stimuli include TGF-β1, mechanical force and matrix stiffness (17).

**Figure 2:**
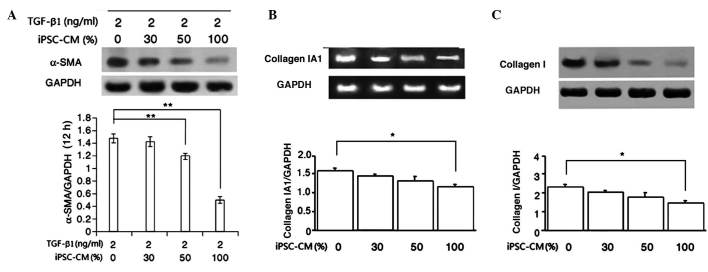
iPSC-CM suppresses fibroblast activation. (A) Protein levels of α-SMA following incubation with iPSC-CM (0, 30, 50 and 100%) and TGF-β1 (2 ng/ml) for 12 h. (B) Expression levels of collagen type Iα1 mRNA following incubation with iPSC-CM (0, 30, 50 and 100%) for 24 h. (C) Protein levels of collagen I following incubation with iPSC-CM (0, 30, 50 and 100%) for 24 h. All values are presented as the mean ± standard error; ^*^P< 0.05, ^**^P<0.01; n=3. iPSC-CM, induced pluripotent stem cells-conditioned medium; α-SMA, α-smooth muscle actin; TGF-β1, transforming growth factor-β1.

To determine whether *in vitro* TGF-β1-induced fibroblast activation may be suppressed by iPSC-CM in the present study, the level of α-SMA expression was assayed using western blot analysis. The results indicated that iPSC-CM significantly suppressed TGF-β1-induced α-SMA expression in a dose-dependent manner in cultured HDFs (P<0.01; Fig. 2A). Additionally, the alterations in collagen type Iα1 expression and type I collagen protein levels were confirmed by RT-qPCR and western blotting. The data demonstrated that the expression levels of collagen type Iα1 mRNA and collagen I protein were reduced, compared with control in 100% iPSC-CM (P<0.05; Fig. 2B and C). These observations suggest that fibroblast activation may be effectively suppressed by iPSC-CM.

### Inhibition of inflammatory cell adhesion on HDFs by iPSC-CM

Monocytes and fibroblasts work together during tissue repair, and fibroblasts regulate the responses of monocytes to ECM-derived matrices (18). Monocytes and lymphocytes are the main cells that induce chronic inflammatory reactions in HS. In addition, THP-1 monocytes are routinely used in monocyte assays (19). Therefore, the adhesion levels of THP-1 and cultured HDFs were examined to identify the inhibition of chronic inflammation by iPSC-CM.

**Figure 3:**
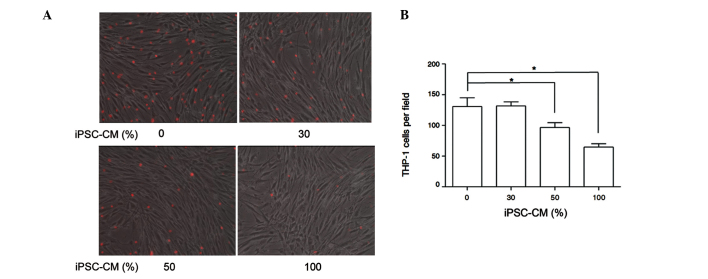
iPSC-CM blocks the adhesion of THP-1 cells. (A) Representative images of adhesion assays. Red spots, calcein AM-labeled adherent THP-1 cells. (B) Quantification of adherent THP-1 cells, n=3. All values are presented as the mean ± standard error, ^*^P<0.05. iPSC-CM, induced pluripotent stem cells-conditioned medium; THP-1, human acute monocytic leukemia cell line; HDFs, human dermal fibroblasts.

*In vitro* adhesion assays of THP-1 and cultured HDFs demonstrated that iPSC-CM significantly reduced the level of adhesion in a dose-dependent manner (Fig. 3). These results suggest that iPSC-CM may suppress fibroblast activation by inducing the recruitment of inflammatory cells and blocking the direct interaction of inflammatory cells and fibroblasts. However, to confirm the attenuated effect of iPSC-CM on the inflammatory response in HS, *in vivo* studies are required.

### iPSC-CM reduces the contractile ability of HDFs in 3D collagen gels

**Figure 4:**
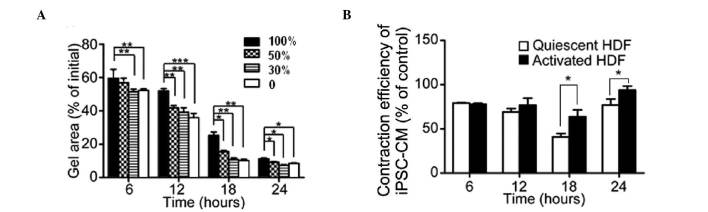
iPSC-CM attenuates the contractile ability of HDFs in 3D collagen gels. (A) Quantification of collagen gel contraction following treatment with iPSC-CM (0, 30, 50 and 100%) for 6, 12, 18 and 24 h; n=3. (B) Contraction efficiency of iPSC-CM on quiescent HDFs and activated HDFs treated with TGF-β1 for 6, 12, 18 and 24 h; n=3. All values are presented as the mean ± standard error. ^*^P<0.05, ^**^P<0.01, ^***^P<0.001. iPSC-CM, induced pluripotent stem cells-conditioned medium; HDFs, human dermal fibroblasts; TGF-β1, transforming growth factor-β1.

Tissue contraction is dynamic and is characterized by intracellular and extracellular events (20). The contraction of HS is not determined by fibroblast properties alone, but is also dependent on the rate and extent of matrix contraction. To evaluate the effect of iPSC-CM on the contractile ability of HDFs, a 3D collagen gel fibroblast contraction assay was performed. Subsequent to treatment with 0, 30, 50 or 100% iPSC-CM for 6, 12, 18 or 24 h, the contraction of the collagen gels was monitored by measuring the gel area. Significant dose-dependent inhibitory effects of iPSC-CM on the contractile ability of HDFs were observed, and as time progressed, the differences between 100% iPSC-CM and the other three groups remained statistically significant (Fig. 4A). Significant time- and dose-dependent inhibitory effects of iPSC-CM on the contractile ability of HDFs were observed (Fig. 4A). Additionally, the contraction efficiency of iPSC-CM was significantly lower in the quiescent HDFs compared with the activated HDFs, at 18 and 24 h (P<0.05; Fig. 4B). This suggests that iPSC-CM may more efficiently prevent alterations in the contractile ability of activated HDFs treated with TGF-β, compared with quiescent HDFs. These observations further support the hypothesis that iPSC-CM is able to suppress fibroblast activation *in vitro*.

## Discussion

HS is a complex and multifactorial fibrotic abnormality that is associated with excessive fibroblast proliferation and collagen synthesis. Previous studies have reported that mutual regulation of chronic inflammation, mechanical force and fibroblast activation leads to the formation of HS in pathological scar formation (16,21,22).

The prolonged existence of chronic inflammation in the active stage of pathological scarring has been investigated in a number of studies (21–23). Histological observations suggest that a large number of macrophages, lymphocytes and mast cells are recruited to the focal site and the early immunological response is important in HS formation (24). Specific cytokines and inflammatory factors, such as TGF-β1 are secreted from these cells and contribute to fibroblast activation and the modulation of the fibroblast phenotype. The results of the current study suggest that iPSC-CM is able to block cell-cell adhesion of inflammatory cells and fibroblasts, while a previous study indicated that fibroblasts can be activated by direct contact with inflammatory cells, such as THP-1 cells (25). Thus, it was concluded that the fibroblast activation suppression by iPSC-CM is partially due to the inhibition of inflammatory cell adhesion.

During the active period of HS, myofibroblasts rooted in irritated fibroblasts are transiently involved in wound repair (26). Under normal conditions, α-SMA (a myofibroblast marker) is not expressed by dermal fibroblasts (27), however, α-SMA expression is observed with mechanical force, which is key in the activation of fibroblasts (28). Conversely, increased numbers of myofibroblasts are responsible for the contractible collagen gels and contraction of wounds during healing (?). In the present study, iPSC-CM significantly reduced the expression of α-SMA in cultured, activated HDFs and notably, produced a significantly greater reduction in the contractile ability of activated HDFs than in quiescent HDFs in 3D collagen gels. From these results, it can be concluded that iPSC-CM may attenuate fibroblast activation via the inhibition of the fibroblast phenotype switch.

Notably, mechanical force has been demonstrated to induce a chronic-like inflammatory state and boost the recruitment of inflammatory cells, including macrophages and lymphocytes (21). Another study indicated that physical force regulates fibrosis through the inflammatory FAK-ERK-MCP-1 pathway (23). It is thus clear that the various etiologies are not independent of each other.

In fibrotic diseases, activated fibroblasts are the main cells involved in the pathogenesis, with abnormal fibroblast activation leading to excessive ECM deposition and increased generation of myofibroblasts. However, the current treatments for fibrotic disorders are unsatisfactory, with an urgent requirement for an effective therapeutic strategy. iPSCs are embryonic-like stem cells that have been demonstrated to have potential in regenerative medicine, while iPSC-CM contains various components, including cytokines and growth factors, which remain to be fully elucidated. The identification of factors that may assist with scar treatment is required and a therapeutic treatment able to prevent abnormal fibroblast activation may be a novel and effective treatment strategy for fibrosis.

In conclusion, the present study indicates that iPSC-CM may be an effective compound in preventing processes leading to HS formation, by attenuating fibroblast activation, blocking inflammatory cell recruitment and adhesion and reducing the contractibility of fibroblasts. However, additional research is required to fully elucidate the biomolecular modulation of the suppressive effect on fibroblast activation by iPSC-CM.

## Figures and Tables

**Table I:** Primer design and product lengths for RT-PCR products.

Gene	Primer	Length (bp)	Tm
COLIA1	5′-AAAGACGGGAGGGCGAGTG-3′		
	5′-GCCATAGGACATCTGGGAAGCAA-3′	242	62
GAPDH	5′-GTCGTGGAGTCTACTGGCGTCTT-3′		
	5′-CAGTCTTCTGAGTGGCAGTGATGG-3′	280	58

RT-PCR, reverse transcription-polymerase chain reaction; bp, base pair; Tm, melting point (the temperature at which the DNA double helix dissociates into single strands); COLIA1, collagen type Iα1.
